# Simultaneous detection of influenza A subtypes of H3N2 virus, pandemic (H1N1) 2009 virus and reassortant avian H7N9 virus in humans by multiplex one-step real-time RT-PCR assay

**DOI:** 10.1186/s40064-016-3733-9

**Published:** 2016-12-01

**Authors:** Dawei Cui, Dejian Zhao, Guoliang Xie, Xianzhi Yang, Zhaoxia Huo, Shufa Zheng, Fei Yu, Yu Chen

**Affiliations:** 1 Department of Laboratory Medicine, First Affiliated Hospital, College of Medicine, Zhejiang University, 79 Qingchun Road, Hangzhou, 310003 China; 2 Key Laboratory of Clinical In Vitro Diagnostic Techniques of Zhejiang Province, Hangzhou, 310003 China

**Keywords:** Influenza A virus, Multiplex real-time RT-PCR, Pandemic (H1N1) 2009 virus, Reassortant avian H7N9 virus, Seasonal H3N2 virus

## Abstract

**Background:**

Influenza A virus is a leading causative pathogen of human acute respiratory infection. Recently, the co-circulation of pandemic (H1N1) 2009 and seasonal H3N2 viruses was reported, and sporadic cases with reassortant avian H7N9 virus are continually reported in China. We aimed to establish a multiplex one-step real-time reverse transcription-polymerase chain reaction (rRT-PCR) assay to simultaneously detect and discriminate FluA subtypes, including human seasonal H3N2 virus, pandemic (H1N1) 2009 virus and reassortant avian H7N9 virus, in one reaction tube.

**Methods:**

Clinical samples, including throat swabs and sputum, were collected from the patients with influenza-like illness (ILIs). Total viral RNA from each sample or viral culture was extracted, and the specific detection of FluA virus and its subtypes was performed using a multiplex rRT-PCR assay.

**Results:**

The limitation of detection (LOD) of the multiplex assay was 5.4 × 10^−2^ 50% tissue culture infective dose (TCID_50_) per reaction or 4.8 × 10^1^ copies per reaction for each virus of the three viruses. For simultaneously detecting the three viruses, the LOD was 1.8 × 10^−2^ TCID_50_ per reaction or 1.6 × 10 copies per reaction for testing the total FluA virus RNA and 5.6 × 10^−2^ TCID_50_ per reaction or 5.1 × 10 copies per reaction for the H3, H1, and H7 genes in one reaction tube. The multiplex assay specifically detected these viruses, and no cross-reaction with other pathogens was found. Moreover, the assay had reliable clinical sensitivity (100%) and valuable clinical specificity (>95%). The detection of FluA with the matrix (M) gene contributed to the further determination of these subtypes, and the Rnase P gene (RP) was considered an internal control to favourably evaluate the quality of the clinical samples.

**Conclusions:**

These findings indicate that the multiplex assay can simultaneously detect and discriminate FluA subtypes with reliable sensitivity and specificity, which is required for the early clinical diagnosis and viral surveillance of patients with FluA infection.

## Background

Influenza A (Flu A) virus is the most widespread pathogen causing human respiratory infections, which is historically associated with annual seasonal epidemics and influenza pandemics worldwide, such as those outbreaks in 1918, 1957, 1968, and 2009 (Cox and Subbarao 2000; Chen et al. 2011; Bedford et al. 2015). The human H1, H2, and H3 subtypes of the FluA viruses generally cause human infections. Recently, pandemic (H1N1) 2009 emerged worldwide in 2009, and the human H3N2 outbreak in 2015 resulted in substantial human infections and deaths (Bedford et al. 2015; Wu et al. 2014; Davlin et al. 2016; Yang et al. 2015). Moreover, sporadic cases or outbreaks of the avian Flu A subtype, such as H5N1, H7N7, H10N8, and H9N2, have displayed direct transmission from domestic poultry and/or wild birds to humans, especially the reassortant avian H7N9 virus from live poultry or avian outbreaks in China in 2013 (Wu et al. 2014; Kudo et al. 2012; Kim et al. 2012; Koopmans et al. 2004; Chen et al. 2013, 2014; Cheng et al. 2011; Ostrowsky et al. 2012; Gao et al. 2013). Most patients infected with these subtypes present similar symptoms with influenza-like illness (ILIs) in the early stages of disease, such as fever, fatigue and/or sore throat, severe acquired pneumonia, and even death.

Recently, the co-circulation of the pandemic (H1N1) 2009 virus and the human H3N2 virus was reported, and patients infected with reassortant avian H7N9 virus have continuously increased in China (Bedford et al. 2015; Davlin et al. 2016; Yang et al. 2015; Chong et al. 2016). Thus, the early detection and diagnosis of the suspected cases is the most effective measure to control and prevent further transmission of these FluA viruses (Chen et al. 2011, 2013; Bedford et al. 2015). Currently, high-throughput and multiplex molecular techniques (polymerase chain reaction, PCR) for the detection of Flu A virus subtypes have been rapidly developed in many laboratories, as previously described (Chen et al. 2011; Kang et al. 2014; World Health Organization 2013). Therefore, we established a multiplex real-time reverse transcription-PCR (rRT-PCR) assay allowing the simultaneous detection and discrimination of the universal Flu A strains, pandemic (H1N1) 2009, human H3N2 and reassortant avian H7N9 subtypes, and the Rnase P gene (RP) served as an internal control to monitor the quality of the clinical samples in one reaction tube.

## Methods

### Primer and probe design

The primers and probe of the FluA virus were designed based on the conserved regions of matrix (M) gene of 85 FluA viruses, and the primers and probes of the human H3 gene of the human H3N2 virus, the human H1 gene of the pandemic (H1N1) 2009, and the H7 gene of the human with reassortant avian H7N9 virus infection were designed based on the highly conserved regions of the HA genes (26 H3N2, 27 H3N2, and 32 H7N9), which are available in the Global Initiative on Sharing Avian Influenza Data (GISAID) and GenBank. The RP gene was used as an internal control to monitor the quality of the clinical specimen. The new primers and probes listed in Table 1 were synthesized by Sangon Biotech (Shanghai) Co., Ltd. (Shanghai, China). The referenced primers and probes from previous studies were also used for comparison with the multiplex assay in this study (Chen et al. 2011; World Health Organization 2013).

**Table 1:** Real-time RT-PCR primers and probes designed and used in this study

Primer and probe sets	Sequences	Gene target	Location (bp)	GenBank accession no.
*Designed and used in this study*				
Flu A forward	GGARTGGCTAAAGACAAGACCAATC	Matrix protein	129–153	
Flu A reverse	GGCATTYTGGACAAAGCGTCTAC	Matrix protein	227–249	KC885959.1
Flu A probe	ROX-AGTCCTCGCTCACTGGGCACGGT-BHQ2	Matrix protein	199–221	
(H1N1) 2009 forward	GGAAAGAAATGCTGGATCTGGTA	Hemagglutinin	822–844	
(H1N1) 2009 reverse	ATGGGAGGCTGGTGTTTATAGC	Hemagglutinin	904–925	KP019929.1
(H1N1) 2009 probe	TAMRA-TGCAATACAACTTGTCARACACCCGAAGG-BHQ2	Hemagglutinin	874–902	
H3 forward	GMAGCAAAGCCTACAGCAACT	Hemagglutinin	269–289	
H3 reverse	GCCGGATGAGGCAACTAGTG	Hemagglutinin	329–348	KP662684.1
H3 probe	HEX-CTTATGATGTGCCGGATTATGCCTCCC-BHQ1	Hemagglutinin	296–322	
H7 forward	GAGGCRATGCAAAATAGAATACAGAT	Hemagglutinin	1510–1535	
H7 reverse	CCGAAGCTAAACCARAGTATCACA	Hemagglutinin	1569–1592	KC885956.1
H7 probe	FAM-ACCCRGTCAAACTAAGCAGCGGYTAYAA-BHQ1	Hemagglutinin	1538–1565	
RP forward	AGATTTGGACCTGCGAGCG	Ribonuclease P	50–68	
RP reverse	GAGCGGCTGTCTCCACAAGT	Ribonuclease P	71–93	NM_006413.4
RP probe	CY5-TTCTGACCTGAAGGCTCTGCGCG-BHQ2	Ribonuclease P	95–114	
*Designed in previous study and used in this study*				
Flu A forward	GACCRATCCTGTCACCTCTGAC	Matrix protein		
Flu A reverse	AGGGCATTYTGGACAAAKCGTCTA	Matrix protein		WHO (2013)(17)
Flu A probe	FAM-TGCAGTCCTCGCTCACTGGGCACG-BHQ1	Matrix protein		
H1 (2009) forward	TGAGATATTCCCCAAGACAAGTTC	Hemagglutinin		
H1 (2009) reverse	TTTGTAGAAGCTTTTTGCTCCAG	Hemagglutinin		Reference (2)
H1 (2009) probe	FAM-TCATGACTCGAACAAAGGTGTAACGG-BHQ1	Hemagglutinin		
H3 forward	ACCCTCAGTGTGATGGCTTCCAAA	Hemagglutinin		
H3 reverse	TAAGGGAGGCATAATCCGGCACAT	Hemagglutinin		Reference (2)
H3 probe	CY5-ACGCAGCAAAGCCTACAGCAACTGT-BHQ2	Hemagglutinin		
H7 forward	AGAAATGAAATGGCTCCTGTCAA	Hemagglutinin		
H7 reverse	GGTTTTTTCTTGTATTTTTATATGACTTAG	Hemagglutinin		WHO (2013)(17)
H7 probe	FAM-AGATAATGCTGCATTCCCGCAGATG-BHQ1	Hemagglutinin		
RP forward	AGATTTGGACCTGCGAGCG	Ribonuclease P		
RP reverse	GAGCGGCTGTCTCCACAAGT	Ribonuclease P		WHO (2013)(17)
RP probe	FAM-TTCTGACCTGAAGGCTCTGCGCG-BHQ1-	Ribonuclease P		

Flu A, universal influenza A virus; H1 (2009), pandemic [H1N1] 2009 virus; H3, seasonal H3N2 virus; H7, reassortant avian H7N9 virus; RP, Rnase P gene. HEX is hexachloro-fluorescein phosphoramidite; BHQ is Black Hole Quencher; ROX is carboxy-X-rhodamine; FAM is a proprietary fluorophore similar to 6-carboxyfluorescein fluorophore; CY5 is blue reporter dye xyanocyline; TAMRA is Carboxytetramethylrhodamine dye. The probe consisted of oligonucleotides with the 5′ reporter dye and the 3′ quencher dye

### Clinical samples

A total of 523 clinical specimens from the patients with ILIs, including 436 throat swabs and 87 sputum specimens, were obtained from March 2014 to November 2015 at the Infection Department, the First Affiliated Hospital, College of Medicine, Zhejiang University. All specimens were kept in viral transport medium supplemented with penicillin (100 U/ml) and streptomycin (100 μg/ml) at −80 °C. The sample from each patient was divided into three tubes. The first was for viral detection, the second was for viral culture, and the last was stored. Informed consent was obtained from all individuals enrolled in this study, and the Ethics Committee of our hospital approved this study.

### Virus isolation and total RNA or DNA extraction

Madin-Darby canine kidney (MDCK) cells were used for isolating the FluA virus by standard methods in a biosafety level-2 (BSL-2) laboratory for human H3N2 virus and pandemic (H1N1) 2009, and a BSL-3 laboratory was used for the reassortant avian H7N9 virus. Each fresh sample was inoculated with MDCK cells. When the cytopathogenic effect (CPE) of the FluA virus infected cells was observed, the cultured cells were harvested and clarified by low speed centrifugation (8000 g/min). The harvested virus was detected by a PCR assay and certified by a sequence assay. The total viral RNA of the individual sample or the viral culture was extracted in 50 μl of the elution buffer using the QIAamp Viral RNA Mini Kit (Qiagen, Valencia, CA) according to the manufacturer’s protocol, and the total DNA of the pathogens was extracted in 100 μl of the elution buffer using the QIAamp DNA Mini Kit (Qiagen, Valencia, CA) according to the manufacturer’s protocol. The samples were stored at −70 °C.

### Multiplex rRT-PCR assay

The multiplex one-step rRT-PCR assay was performed by a one-step PrimeScript^®^ RT-PCR reagent (Takara, Dalian, China). The final optimised 50 μl reaction mixture consisted of 5 μl of RNA, 25 μl of 2× One Step RT-PCR Buffer, 1 μl of TaKaRa EX Taq HS (5 U/μl), 1 μl of PrimeScript RT Enzyme Mix, 1 μl of forward primer (40 μM), 1 μl of reverse primer (40 μM), 0.5 μl of probe (20 μM) for each virus, 0.8 μl of RP forward primer (40 μM), 0.8 μl of RP reverse primer (40 μM), 0.4 μl of RP probe (20 μM), and 6 μl of RNase-free water. The thermal cycling parameters were as follows: cDNA synthesis at 42 °C for 30 min; denaturation at 95 °C for 5 min; and 40 cycles at 95 °C for 10 s; and 60 °C for 45 s. The fluorescence was recorded at 60 °C on an ABI 7500 real-time PCR system (Applied Biosystems, Foster City, CA).

### Sensitivity and specificity

The titers of the three viruses were calculated by determining the 50% tissue culture infective dose (TCID_50_). The sensitivity of the multiplex assay was determined by testing tenfold serial dilutions of the human H3N2 virus, the pandemic (H1N1) 2009 or reassortant avian H7N9 virus, or a mixture of the three viruses. Additionally, the sensitivity of the multiplex assay was also evaluated using tenfold serial dilutions of the RNA transcripts that were transcribed in vitro by a recombinant pGEM^®^-Easy plasmid (Promega, Shanghai, China) and the RiboMAXTM Large Scale RNA Production System-SP6 (Promega, Madison, Wisconsin, USA) according to the manufacturer’s protocol, as described previously (Chen et al. 2011; Kang et al. 2014).

The specificity of the multiplex assay was evaluated using the three viruses and their mixtures as well as a panel comprised of 18 other common respiratory pathogens (Seasonal H1N1 virus, influenza B virus, avian H5N1, avian H9N2, respiratory syncytial virus [types A and B], Enterovirus 71, Coxsackie A16, human rhinovirus, *Mycoplasma pneumoniae, Klebsiella pneumoniae, Acinetobacter baumannii, Pseudomonas aeruginosa, Staphylococcus aureus, Candida albicans, Neisseria meningitides,* human parainfluenza virus-2, and human adenovirus) at high concentrations (>10^4^ TCID50, copies, or cells/ml). First, the three viruses and their mixtures were tested by the multiplex assay. Second, each nucleic acid (or their mixtures) of the 18 pathogens was tested by the multiplex assay. Third, the multiplex assay was used to test one or the three viruses, including one (or their mixtures) of the 18 pathogens. All of the pathogens were tested in triplicate by three independent runs using the multiplex assay and the rRT-PCR assay released by WHO and our previous study (Chen et al. 2011; World Health Organization 2013).

### Detection of clinical specimens using multiplex rRT-PCR assay

The clinical sensitivity and specificity were calculated using 523 clinical specimens that were blindly detected and compared using the multiplex assay, the referenced methods and tissue culture. For the positive, suspected or different results, the partially amplified DNA products of the positive sample were cloned into the pMD18T vector (Takara, Dalian, China) that was transformed into *Escherichia coli*, and their sequences were verified by a genomic sequence analysis performed at Sangon Biotech Co., Ltd. (Shanghai, China).

## Results

### Multiplex rRT-PCR assay development and optimisation

The M gene and the HA gene sequences of 26 seasonal H3N2, 27 pandemic (H1N1) 2009, and 32 reassortant avian H7N9 viruses available from GISAID and GenBank were aligned and compared, respectively. A conserved region was selected to design the primer and probe sets for the M gene from these FluA subtypes, and a conserved region was selected for the H3, H1 and H7 genes. These primers-probe sets were used to test the RNA of the corresponding FluA subtype at primer concentrations of 0.2, 0.4, 0.8, 1.6, and 3.2 μM and probe concentrations ranging from 0.2 to 3.2 μM in a 50 μl reaction volume. The optimal concentration of the primers and the probe sets was 0.4 and 0.2 μM, respectively, for the M, H1, H3, H7, and RP genes (data not shown).

The final optimised 50 μl reaction mixture consisted of 5 μl of RNA, 25 μl of 2× One Step RT-PCR Buffer, 1 μl of TaKaRa EX Taq HS (5 U/μl), 1 μl of PrimeScript RT Enzyme Mix, 1 μl of forward primer (40 μM) and 1 μl of reverse primer (40 μM), 0.5 μl of probe (20 μM) for each virus, 0.8 μl of RP forward primer (40 μM), 0.8 μl of RP reverse primer (40 μM), 0.4 μl of RP probe (20 μM), and 6 μl of RNase-free water.

### Sensitivity and specificity

The sensitivities and specificities of the multiplex rRT-PCR assay as well as those of the referenced rRT-PCR methods were evaluated by analysing the serial dilutions of the three viruses and their related RNA transcripts. In our study, the original concentration of each of the three subtypes of FluA viruses (pandemic [H1N1] 2009, human H3N2 and reassortant avian H7N9 virus) was 5.4 × 10^3^ 50% tissue culture infective dose (TCID_50_)/ml or 4.8 × 10^6^ copies/ml. The limitation of detection (LOD) was 5.4 × 10^−2^ TCID_50_ per reaction or 4.8 × 10 copies per reaction for each virus subtype.

For the three mixed virus specimens, the final titer of each virus in 200 μl of the mixed virus specimens was 1.8 × 10^3^ 50% TCID_50_/ml, and the original mixture of the RNA transcripts for each FluA subtype was 1.6 × 10^6^ copies/ml. The LOD of the multiplex assay for simultaneous detecting the three mixed viruses was approximately 1.8 × 10^−2^ TCID_50_ per reaction or 1.6 × 10 copies per reaction for total FluA virus and 5.6 × 10^−2^ TCID_50_ per reaction or 5.1 × 10 copies per reaction for the H3, H1, and H7 genes in one reaction tube.

**Fig. 1:**
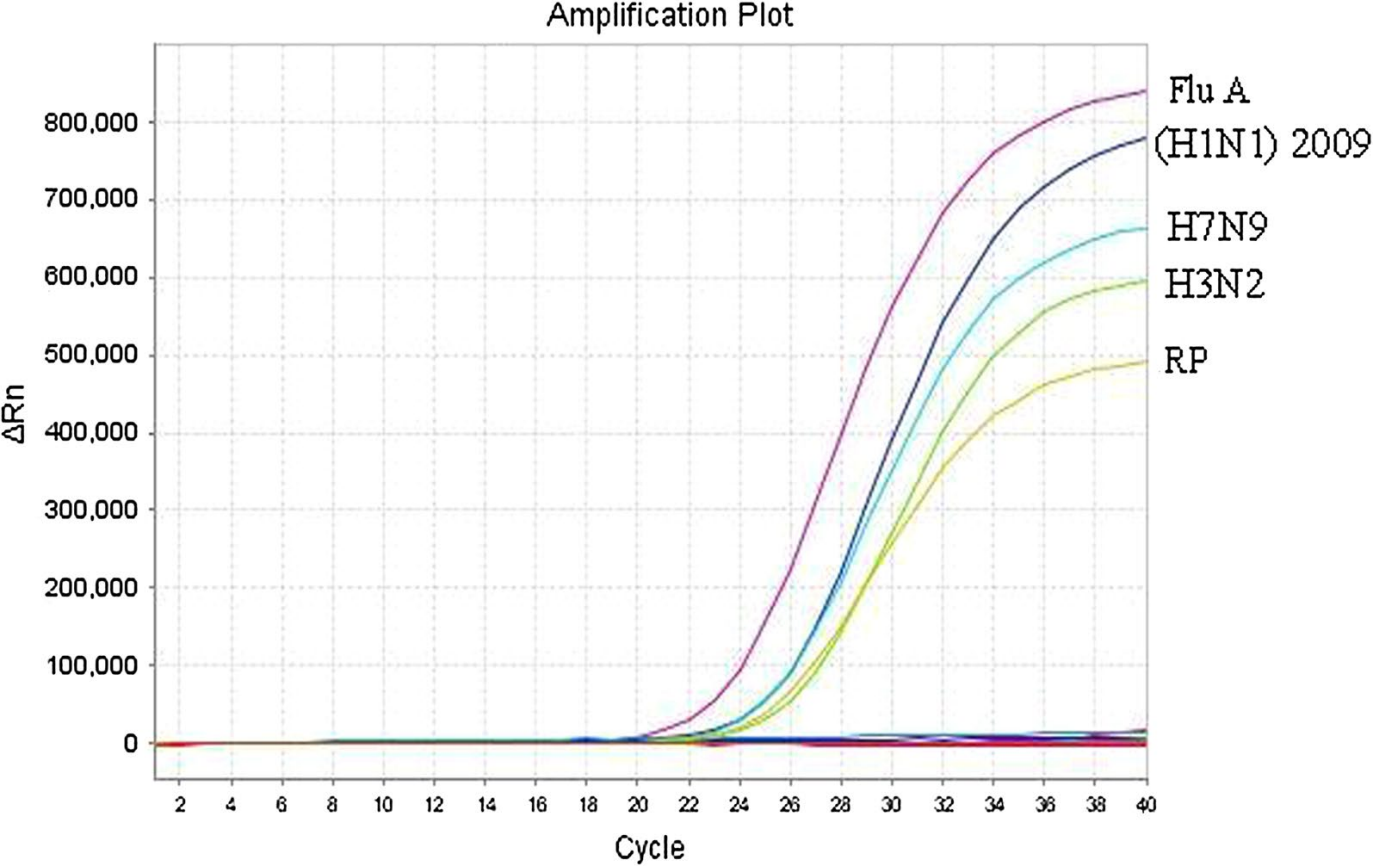
Specificity of the multiplex rRT-PCR assay for pandemic (H1N1) 2009, H3N2, and reassortant avian H7N9 viruses. Signals from the RNA samples extracted from human pandemic (H1N1) 2009, seasonal H3N2, reassortant avian H7N9 virus (A/Zhejiang/DTID-ZJU01/2013), the clinical samples with negative FluA virus, and RP gene

As for the multiplex assay specificity, the multiplex assay specifically detected the three subtypes of FluA virus. We did not observe cross-reaction among the three types of viruses (pandemic [H1N1] 2009, human H3N2 and reassortant avian H7N9 virus) or among the panel comprising the 18 other common respiratory pathogens at high concentrations (>10^4^ TCID50, copies, or cells/ml), excluding the FluA gene in this study (Fig. 1). Additionally, the referenced rRT-PCR assay had a similar specificity for the three types of viruses.

### Detection of clinical specimens

To further evaluate the clinical sensitivity and specificity, each of the 523 clinical samples, including 436 throat swabs and 87 sputum specimens from the patients with ILIs, was inoculated into MDCK cells for influenza A virus isolation. The results showed that 125 samples were positive for FluA virus, including 28 pandemic (H1N1) 2009, 88 human H3N2 and 9 reassortant avian H7N9 samples. Compared with the cell culture, the analyses of the 523 samples by the multiplex rRT-PCR assay and referenced methods in parallel showed that 144 specimens were positive for FluA, including 36 specimens with pandemic (H1N1) 2009 virus, 101 specimens with H3N2 virus, and 9 specimens with reassortant avian H7N9 virus (Table 2). Moreover, the results showed that the detection sensitivity for FluA, pandemic (H1N1) 2009, H3N2, and reassortant avian H7N9 virus was 100% (95% confidence interval [CI] 100–100%). The specificity was 95.2% (95% CI 93.1–97.3%) for FluA virus, 98.4% (95% CI 97.3–99.5%) for pandemic (H1N1) 2009 virus, 97.0% (95% CI 95.4–98.6%) for H3N2 virus, and 100% (95% CI 100–100%) for reassortant avian H7N9 virus. Additionally, the results by the multiplex assay were fully comparable with the referenced rRT-PCR methods in this report.

**Table 2:** Sensitivity and specificity of multiplex rRT-PCR assay for 523 clinical specimens

Virus	Culture		Multiplex		Reference		Sens. (%)	Spec. (%)
	+	−	+	−	+	−	(95% CI)	(95% CI)
Flu A	125^a^	398	144	379	144	379	100 (100–100)	95.2 (93.1–97.3)
H1N1(2009)	28^b^	495	36 ^d^	487	36^e^	487	100 (100–100)	98.4 (97.3–99.5)
H3N2	88^c^	435	101^d^	422	101^e^	422	100 (100–100)	97.0 (95.4–98.6)
H7N9	9	514	9	514	9	514	100 (100–100)	100 (100–100)
RP	–	523	523	–	523	–	100 (100–100)	100 (100–100)

Values refer individually to the virus culture

*Sens.* sensitivity, *Spec.* specificity, *95% CI* 95% confidence interval [CI]

^a^Nineteen negative samples by cell culture consisted of eight pandemic H1N1 (2009) ^b^ and eleven H3N2 virus ^c^ by PCR and genomic sequence analysis. Indeterminate results were excluded from sensitivity and specificity calculations

^d^Two patients with H1N1(2009) virus-H3N2 virus coinfections, one was identified in throat swab specimen from patient with kidney transplantation history, the other was detected in sputum specimen from patient with multiple myeloma history. However, there was only H3N2 virus cultured in the two patients specimens, respectively

## Discussion

FluA virus is a negative-sense, single-stranded RNA virus that can be further classified into various subtypes according to two surface glycoproteins with hemagglutinin (HA) and neuraminidase (NA) (Cox and Subbarao 2000; Chen et al. 2011). Sixteen H subtypes (H1–H16) and nine N subtypes (N1–N9) have been found in avian species, while the H17N10 and H18N11 subtypes have emerged in bats (Bedford et al. 2015; Wu et al. 2014; Fan et al. 2014). Human infections with FluA viruses are commonly associated with the human H1, H2, and H3 subtypes that can cause outbreaks of FluA virus infection, such as the seasonal H3N2 virus and the pandemic (H1N1) 2009 virus (Cox and Subbarao 2000; Chen et al. 2011; Bedford et al. 2015; Davlin et al. 2016; Yang et al. 2015). However, sporadic cases or outbreaks of the avian FluA subtypes H5N1, H7N3, H10N8, and H9N2, and the reassortant avian H7N9 virus have resulted in the direct transmission from avians to humans and have caused many deaths in humans (Kudo et al. 2012; Kim et al. 2012; Koopmans et al. 2004; Chen et al. 2013, 2014; Cheng et al. 2011; Ostrowsky et al. 2012; Gao et al. 2013). Most patients infected with these subtypes have mild symptoms during the early stages of the disease, such as acute respiratory infections associated with fever and/or sore throat, with or without fatigue symptoms; however, the patients with H5N1 virus and reassortant avian H7N9 virus have a high mortality rate (Cheng et al. 2011, 2013, 2014; Ostrowsky et al. 2012; Gao et al. 2013; Chong et al. 2016; Kang et al. 2014).

Currently, the pandemic (H1N1) 2009 virus and the human H3N2 virus infections are reported worldwide, and sporadic cases of reassortant avian H7N9 virus infection are continuously documented in China (Bedford et al. 2015; Davlin et al. 2016; Yang et al. 2015; Chong et al. 2016). It is well known that PCR, which is conducted in many laboratories, is highly sensitive and specific for the detection of FluA virus and its subtypes (Cox and Subbarao 2000; Chen et al. 2011). High-throughput and multiplex rRT-PCR assay for the detection of Flu A virus subtypes has rapidly been established in many laboratories (Chen et al. 2011; Kang et al. 2014; World Health Organization 2013). However, a multiplex rRT-PCR assay for the simultaneous detection of the human H3N2 virus, the pandemic (H1N1) 2009 virus and the reassortant avian H7N9 virus has not been reported. Therefore, there is an urgent need to establish a multiple and specific multiplex rRT-PCR assay, which is the most effective measure, for the early detection and confirmation of human H3N2 virus, pandemic (H1N1) 2009 virus and reassortant avian H7N9 virus to reduce the morbidity and mortality rates of patients with these viruses. Here, we developed a multiplex one-step rRT-PCR assay that simultaneously detected and discriminated the universal Flu A virus, the pandemic (H1N1) 2009, the human H3N2 and the reassortant avian H7N9 subtypes, and the Rnase P gene (RP) as an internal control in one reaction tube. The multiplex rRT-PCR assay had a reliable sensitivity and specificity for the three viruses compared with the referenced rRT-PCR assay. Additionally, in this study, the LOD of the multiplex assay for the mixed viruses was slightly low but was high for the FluA virus compared to that of a single subtype of the FluA virus. For detecting a single subtype of virus, the LOD of the multiplex assay was the same as the referenced rRT-PCR assay. These results indicated that the simultaneous amplification of two or more types of virus may cause a lower LOD in the multiplex assay when using multiplex primers and probes in one reaction. Moreover, the pandemic (H1N1) 2009, human H3N2 and reassortant avian H7N9 viruses belong to the Flu A virus, and the slightly higher sensitivity of the multiplex assay for the FluA virus may be due to the simultaneous amplification of the three types of Flu A virus in one reaction.

The “gold standard” for the diagnosis of viral infection is considered to be tissue culture (Chen et al. 2013; Fan et al. 2014). To further evaluate the clinical sensitivity and specificity of the multiplex assay, each of the 523 clinical samples from the patients with ILIs was inoculated into MDCK cells for influenza A virus isolation, and the three viruses were detected by the multiplex assay and the referenced methods. The results of the multiplex assay were fully comparable with the referenced rRT-PCR methods in this report. However, there were two specimens with pandemic (H1N1) 2009 virus-H3N2 virus coinfections, and only H3N2 virus was cultured in the two specimens. Moreover, the two samples with coinfections were also confirmed by the multiplex assay, the referenced methods, and the genomic sequence analysis. Therefore, the two samples with coinfections were included in the sensitivity and specificity calculations. Additionally, the cycle threshold (Ct) values were all <32.0 using the multiplex assay and the referenced methods for each of the 523 clinical samples, including the RP target gene. In this study, 19 specimens were negative by the viral isolations but were positive by the PCR results, showing high Ct values (28.5–30.6), which indicated a low viral concentration. The discrepancy may have resulted from various factors. With a very low concentration of virus in the specimens, it can be difficult to generate a viral isolation, or it may be possible that inhibitory components in the specimens prevent viral isolation in cell culture. Additionally, the competitive growth of two viruses in one sample may result in the isolation of only one virus in cell culture. To verify the positive results amplified by the multiplex assay, 12 positive samples with the H3N2 virus, 8 positive samples with the pandemic (H1N1) 2009 virus and 5 positive samples with the reassortant avian H7N9 virus were randomly selected to analyse their HA genomic sequences that were confirmed by the Sangon Biotech Co., Ltd (Shanghai, China). These results strongly indicated that the multiplex rRT-PCR assay in the study was completely in accordance with the assay systems previously described (Chen et al. 2011; Kang et al. 2014; World Health Organization 2013; Fan et al. 2014; Nie et al. 2013; Kuo et al. 2014).

Our study had several limitations. First, the multiplex rRT-PCR assay only evaluated three viruses and 18 other common respiratory pathogens. However, more respiratory pathogens should be enrolled in this study. Secondly, some important information (such as the drugs administered) should be included, as certain drugs may affect the result of the detection and the tissue culture. Finally, the clinical sample population was relatively small in this study.

## Conclusions

In conclusion, a sensitive and specific multiplex one-step rRT-PCR assay was developed for the early detection of most influenza A viruses and subtypes, including pandemic (H1N1) 2009, H3N2, and reassortant avian H7N9 virus, in clinical samples from patients with ILIs. This multiplex assay will provide a rapid and easy method for clinical diagnostics and viral surveillance of influenza A and its subtypes.

## Abbreviations

FluA: influenza A virus
rRT-PCR: real-time reverse transcription-polymerase chain reaction
ILIs: influenza-like illness
LOD: limitation of detection
TCID_50_: 50% tissue culture infective dose
M gene: matrix gene
HA: hemagglutinin
NA: neuraminidase
RP gene: Rnase P gene
GISAID: Global Initiative on Sharing Avian Influenza Data
MDCK: Madin-Darby canine kidney cell
BSL-2: biosafety level-2
CPE: cytopathogenic effect
